# Expression of LAG-3 defines exhaustion of intratumoral PD-1^+^ T cells and correlates with poor outcome in follicular lymphoma

**DOI:** 10.18632/oncotarget.18251

**Published:** 2017-05-29

**Authors:** Zhi-Zhang Yang, Hyo Jin Kim, Jose C. Villasboas, Ya-Ping Chen, Tammy Price-Troska, Shahrzad Jalali, Mara Wilson, Anne J. Novak, Stephen M. Ansell

**Affiliations:** ^1^ Division of Hematology and Internal Medicine, Mayo Clinic, Rochester, MN, USA; ^2^ Division of Oncology/Hematology and Internal Medicine, College of Medicine, National Cheng Kung University and Hospital, Tainan, Taiwan

**Keywords:** follicular lymphoma, LAG-3, T-cell exhaustion, PD-1, immune checkpoint

## Abstract

Exhausted T-cells in follicular lymphoma (FL) typically express PD-1, but expression of PD-1 is not limited to exhausted cells. Although expected to be functionally suppressed, we found that the population of intratumoral PD-1^+^ T cells were predominantly responsible for production of cytokines and granules. This surprising finding prompted us to explore the involvement of LAG-3 to specifically identify functionally exhausted T cells. We found that LAG-3 was expressed on a subset of intratumoral T cells from FL and LAG-3^+^ T cells almost exclusively came from PD-1^+^ population. CyTOF analysis revealed that intratumoral LAG-3^+^ T cells were phenotypically heterogeneous as LAG-3 was expressed on a variety of T cell subsets. In contrast to PD-1^+^LAG-3^-^ cells, intratumoral PD-1^+^LAG-3^+^ T cells exhibited reduced capacity to produce cytokines and granules. LAG-3 expression could be substantially upregulated on CD4^+^ or CD8^+^ T cells by IL-12, a cytokine that has been shown to induce T-cell exhaustion and be increased in the serum of lymphoma patients. Furthermore, we found that blockade of both PD-1 and LAG-3 signaling enhanced the function of intratumoral CD8^+^ T cells resulting in increased IFN-γ and IL-2 production. Clinically, LAG-3 expression on intratumoral T cells correlated with a poor outcome in FL patients. Taken together, we find that LAG-3 expression is necessary to identify the population of intratumoral PD-1^+^ T cells that are functionally exhausted and, in contrast, find that PD-1^+^LAG-3^-^ T cells are simply activated cells that are immunologically functional. These findings may have important implications for immune checkpoint therapy in FL.

## INTRODUCTION

Follicular lymphoma (FL) is characterized by the presence of a significant number of T cells in the tumor microenvironment that have a substantial impact on anti-tumor immunity and patient outcome [1, 2]. Persistent presence of tumor antigens chronically stimulates intratumoral T cells, creating a scenario similar to chronic infections. This scenario usually induces T-cell exhaustion and exhausted T cells exhibit reduced differentiation, proliferation, and effector function. Our group and others have shown that T-cell exhaustion is present in lymphoma patients and results in T-cell inhibition and loss of effective immune function [3–6].

It has been well known that PD-1 is a marker of T-cell exhaustion and plays a crucial role in the induction of T cell exhaustion [7, 8]. However, PD-1 is also expressed on T cells present within the follicles of secondary lymphoid organs, and some PD-1^+^ T cells have been identified as follicular helper T (T_FH_) cells [9–11]. Besides PD-1^+^ T_FH_ cells, other intratumoral T-cells express PD-1 and not all PD-1^+^ cells seem to be exhausted. In an era when therapies targeting PD-1 to reverse immune exhaustion are commonly used, it is important to determine which PD-1^+^ cells in the tumor are truly exhausted. To better define the population of exhausted T-cells in FL, we studied other molecules associated with T-cell exhaustion [12, 13] and identified the expression of LAG-3 as important in identifying truly exhausted T-cells.

LAG-3 (CD223) is a member of the immunoglobulin superfamily and is expressed on a variety of cell types including T cells, NK cells, B cells and dendritic cells. LAG-3 is generally absent on resting T cells and is upregulated on activated T cells. By interacting with its ligand, MHC class II, LAG-3 plays a negative regulatory role and suppresses T cell function. Studies have found that CD4 T cells exhibit increased proliferation and enhanced cytokine production when LAG-3 is blocked *in vitro* [14, 15]. Furthermore, it has been shown that LAG-3 is differentially expressed on both natural and induced regulatory T cells (T_reg_) and is required for maximal T_reg_ function [16].

In this study, we determined the expression and function of LAG-3 in FL, assessed the role of LAG-3 in contributing to exhaustion of PD-1^+^ T cells, and tested whether targeting both PD-1 and LAG-3 signaling reverses T cell exhaustion in FL.

## RESULTS

### The PD-1^+^ T population is expanded and functionally active in FL

**Figure 1:**
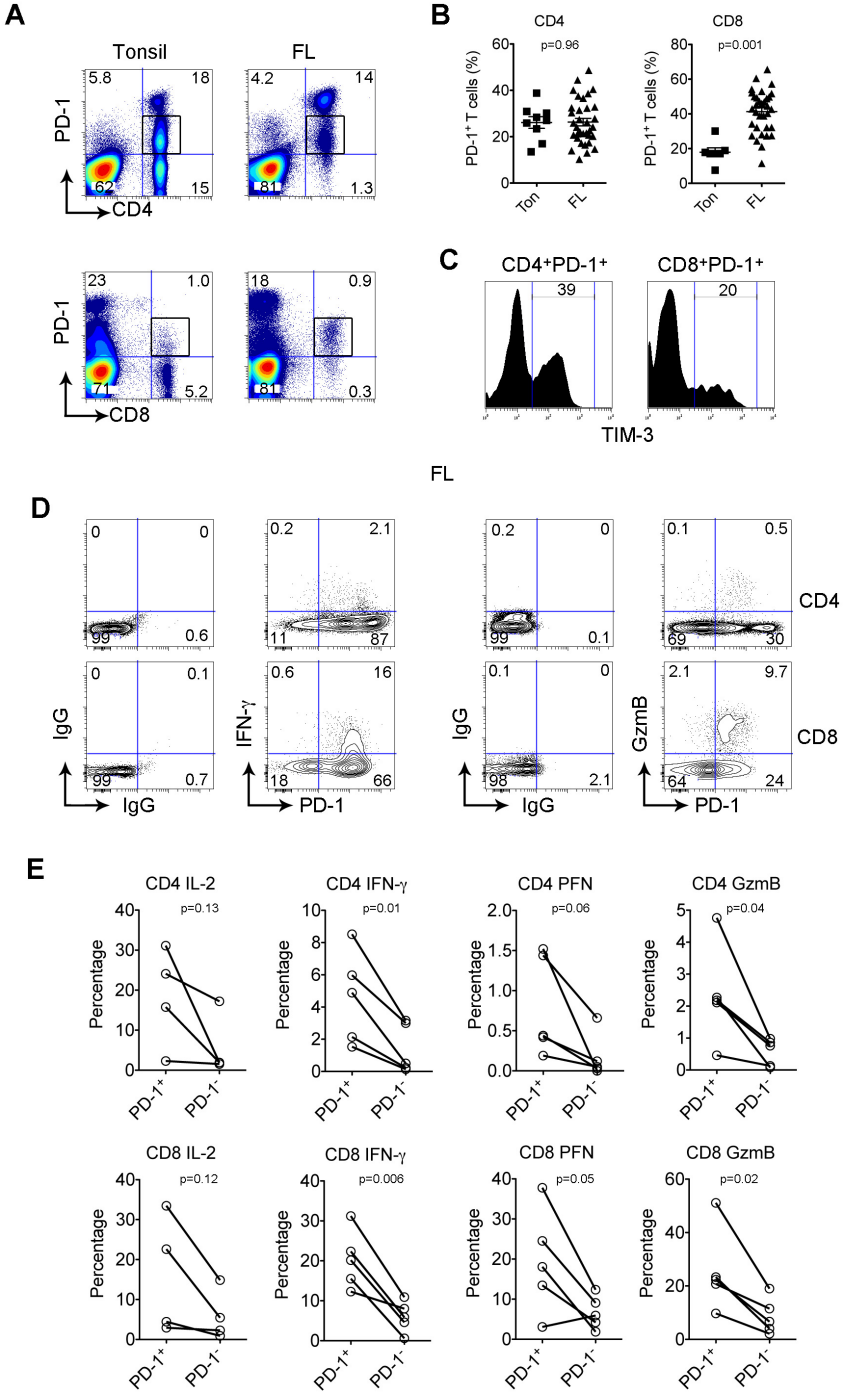
PD-1^+^ T population is expanded and functionally active in FL **(A)** PD-1 expression on CD4^+^ or CD8^+^ T cells from biopsy specimens of a FL patient (FL) and tonsil (Ton). Box is to indicate a PD-1^+^ T population exists in both the CD4^+^ and CD8^+^ subsets. **(B)** Graphs showing percentages of PD-1^+^ CD4^+^ or CD8^+^ T cells from tonsil and FL. **(C)** TIM-3 expression by PD-1^+^ CD4^+^ or CD8^+^ T cells. **(D)** IFN-γ and granzyme B (GzmB) on PD-1^+^CD4^+^ or CD8^+^ T cells from lymph nodes of FL patients. Isotype control staining was performed to determine PD-1^+^ T cells. **(E)** Graph summarizes percentages of IL-2, IFN-γ, perforin (PFN) and GzmB by PD-1^+^ and PD-1^-^ in CD4^+^ and CD8^+^ T cells.

PD-1 is usually absent on resting T cells and induced by activation. In secondary lymphoid organs such as lymph nodes (LN) and tonsils (Ton), we had previously shown that PD-1 has a unique expression pattern with a bright immunohistochemical staining in cells in follicles and dim staining in cells outside follicles [5]. We had found that the PD-1^high^ cells were only present in the CD4^+^ T cell population and were absent from the CD8^+^ T cell population, and their phenotype is that of CD4^+^ T_FH_ T cells [5]. In contrast, we had also shown that the remaining PD-1^+^ cells, that typically expressed lower levels of PD-1 and were present between the malignant follicles, had an exhausted phenotype and lacked normal immune function. To now assess whether all of these remaining PD-1^+^ cells were in fact exhausted or whether only a subset of cells were, we focused on the cells expressing low levels of PD-1 and confirmed that these PD-1^+^ T cells exist in both the CD4^+^ and CD8^+^ subsets (Figure 1A). We then determined whether these cells are more prevalent in FL than in normal tonsil or lymph nodes. Although there was no statistical difference of frequency of CD4^+^PD-1^+^ T cells between tonsil and lymphoma patients, we did find that the numbers of CD8^+^PD-1^+^ T cells were significantly higher in lymphoma tissues than tonsils. PD-1^+^ T cells accounted for approximately 41.35% (range: 11.5%-65.5%, n=33) of CD8^+^ T cells in FL specimens compared to 17.95% (range: 7.58%-30.1%, n=8, p<0.001) of CD8^+^ T cells in tonsil tissues (Figure 1B). However, only a subset of both CD4 and CD8 PD-1^low^ T cells coexpressed TIM-3, a second exhaustion marker (Figure 1C), suggesting that not all PD-1^+^ cells are exhausted.

To test whether all PD-1^+^ T cells in FL display reduced immune function, we measured the capacity of PD-1^+^ T cells to produce cytokines (IL-2 and IFN-γ) and granules (perforin (PFN) and granzyme B (GzmB)). As shown in Figure 1D, we gated on PD-1 T cells and to our surprise observed that cytokines and granules were mainly produced by PD-1^+^ T cells instead of the PD-1^-^ T cell population. Furthermore, we found that the vast majority of IFN-γ- or GzmB-producing cells were CD4^+^ or CD8^+^ PD-1^+^ T cells. This was confirmed by analyzing multiple samples (n=5, Figure 1E). Furthermore, the percentages of cytokine- and granule-producing T cells were significantly higher from the PD-1^+^ than the PD-1^-^ subset in both CD4^+^ and CD8^+^ T cell population (Figure 1E). These results strongly suggest that, instead of a decline in function, cells within the PD-1^+^ population remain functionally active, and may be even more functionally active than PD-1^-^ T cells.

### LAG-3 is primarily expressed on intratumoral PD-1^+^ T cells in FL

**Figure 2:**
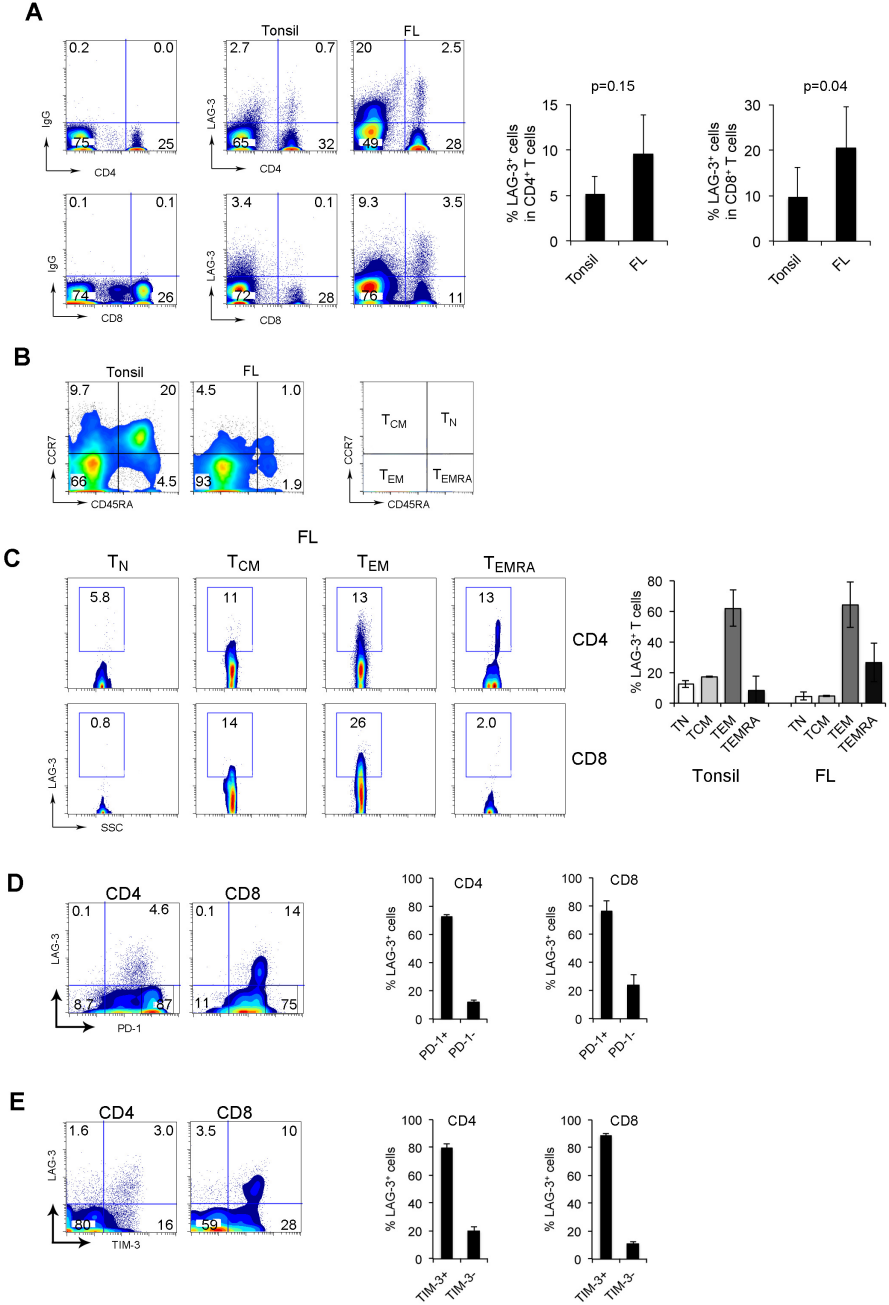
LAG-3 is primarily expressed on intratumoral PD-1^+^ T cells in FL **(A)** LAG-3 expression on CD4^+^ or CD8^+^ T cells from tonsils and FL specimens. The right graphs summarize percentages of LAG-3^+^CD4^+^ or CD8^+^ T cells from tonsils and FL. **(B)** CD45RA and CCR7 expression on CD3^+^ T cells from tonsils and FL specimens. T_N_: naïve T cells (CD45RA^+^CCR7^+^), T_CM_: central memory (CD45RA^-^CCR7^+^), T_EM_: effector memory (CD45RA^-^CCR7^-^) and T_EMRA_: terminally-differentiated T cells (CD45RA^+^CCR7^-^). **(C)** LAG-3 expression by CD4^+^ or CD8^+^ T cell subsets. The right graph summarizes percentages of LAG-3^+^ T cells in T cell subsets of T_N_, T_CM_, T_EM_ and T_EMRA_. (n=5 for tonsils, n=3 for FL patients). **(D-E)** Co-expression of LAG-3 and PD-1 **(D)** or TIM-3 **(E)** in CD4^+^ or CD8^+^ T cells. The right graphs show percentage of LAG-3^+^ T cells in subsets of PD-1 or TIM-3 T cells (n=6).

It has recently been shown that LAG-3 is important in T cell exhaustion and synergistically impacts T cell function with PD-1. To explore the potential role of LAG-3 in better defining exhausted PD-1^+^ T-cells, we determined its expression in biopsy specimens from FL patients. As shown in Figure 2A, LAG-3 expression was negligible on CD4^+^ or CD8^+^ T cells from peripheral blood, but quite visible on CD4^+^ or CD8^+^ T cells from tonsils. However, LAG-3 was highly expressed on CD4^+^ or CD8^+^ T cells from lymphoma biopsies. LAG-3^+^ T cells accounted for 4.96% and 8.9% of CD4^+^ or CD8^+^ T cells from tonsils (n=6) and 8.7% or 20% of CD4^+^ or CD8^+^ T cells from FL patients (n=8), respectively (Figure 2A).

To further define LAG-3^+^ T cells, we assessed the differentiation status of LAG-3^+^CD4^+^ or CD8^+^ T cells from tonsil and FL tumors. Subsets of naïve (T_N_), central memory (T_CM_), effector memory (T_EM_) and terminally-differentiated (T_EMRA_) cells were defined by co-expression of CD45RA and CCR7. While detectable in low numbers in lymphoma tissue, T_N_ cells were present in tonsil tissue at a substantially higher frequency (Figure 2B). We found that the majority of LAG-3^+^ T cells were T_EM_ (CD45RA^-^CCR7^-^) cells in both tonsil and lymphoma tissues. Compared to lymphoma tissues, tonsils had a significant higher number of T_N_ (CD45RA^+^CCR7^+^) or T_CM_ (CD45RA^-^CCR7^+^) cells that expressed LAG-3 (Figure 2C). However, we observed that intratumoral LAG-3^+^ T cells expressed TEMRA markers (CD45RA^+^CCR7^-^), suggesting a terminal differentiation status of LAG-3^+^ T cells in lymphoma.

To determine whether LAG-3 plays a role in further defining PD-1-mediated T-cell exhaustion, we measured expression of LAG-3 on PD-1-expressing T cells. As shown in Figure 2D, LAG-3 was coexpressed with PD-1 and almost exclusively expressed on intratumoral PD-1^+^ T cells. In CD8^+^ T cells in which PD-1 is expressed at a low intensity, co-expression of PD-1 and LAG-3 was observed and almost all LAG-3^+^ T cells were PD-1^+^ cells (Figure 2D). In CD4^+^ T cells in which PD-1 is expressed at both high and low intensity, co-expression of PD-1 and LAG-3 was observed only in PD-1^low^ T cells while PD-1^high^ T cells lack LAG-3 expression (Figure 2D). PD-1^+^ LAG-3^+^ T cells accounted for approximately 73% (n=5) and 76% (n=6) of CD4^+^ and CD8^+^ LAG-3-expressing cells, respectively (Figure 2D).

We have previously shown that TIM-3 is co-expressed with PD-1 and plays an important role in T-cell exhaustion in FL. We next explored whether LAG-3 was co-expressed with TIM-3 in intratumoral T cells in lymphoma. As shown in Figure 2E, LAG-3 was co-expressed with TIM-3 and the vast majority of LAG-3^+^ T cells were TIM-3^+^ T cells. TIM-3^+^LAG-3^+^ T cells accounted for approximately 80% (n=5) and 89% (n=5) of CD4^+^ and CD8^+^ LAG-3-expressing cells, respectively (Figure 2E). Taken together, the co-expression of LAG-3 with PD-1 and TIM-3 formed a defined population of PD-1^+^TIM-3^+^LAG-3^+^ T cells in the tumor microenvironment of FL.

### Intratumoral LAG-3^+^ T cells are phenotypically heterogeneous

**Figure 3:**
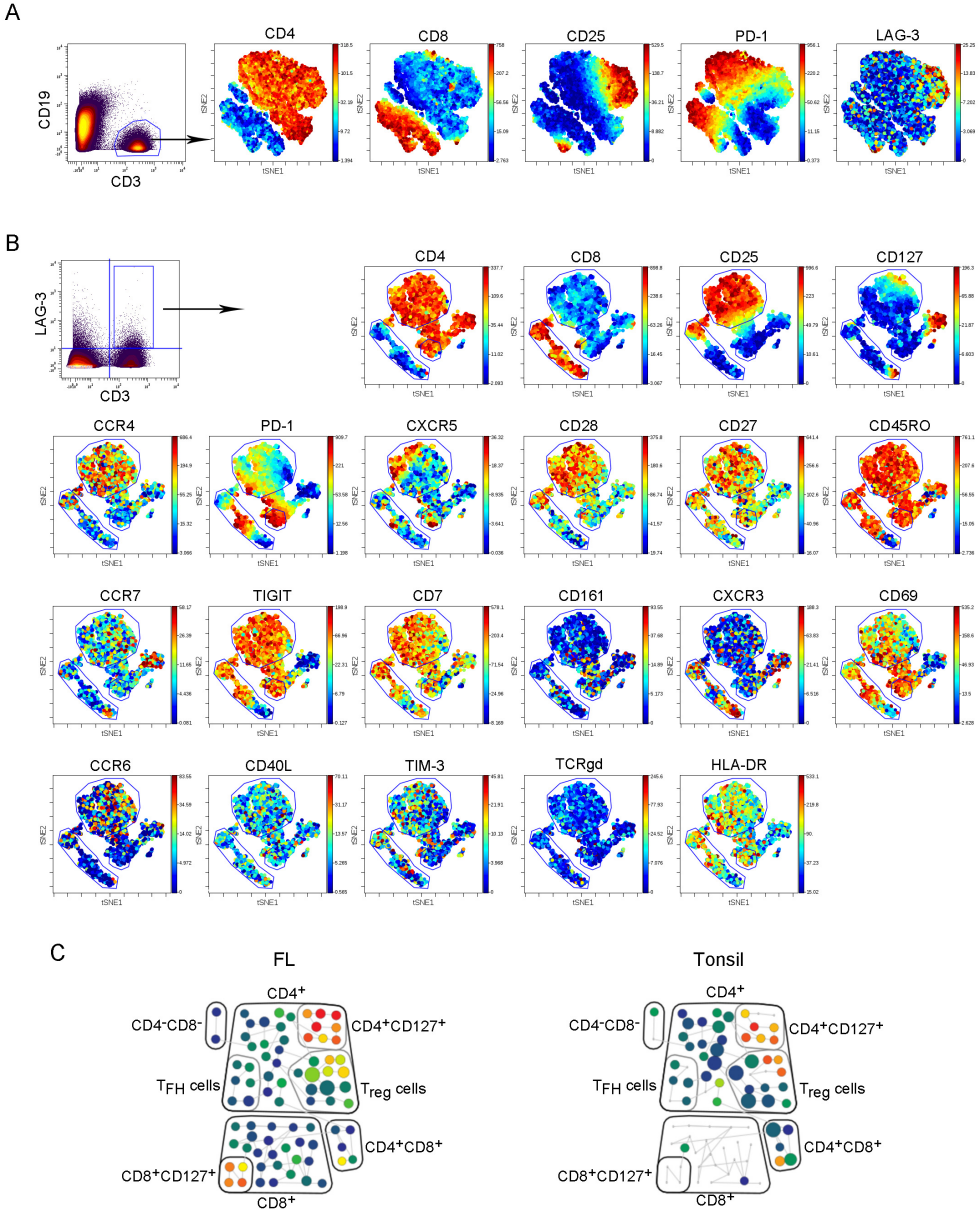
Intratumoral LAG-3^+^ T cells are phenotypically heterogeneous **(A-B)** The viSNE map of a representative patient sample (n=28) showing expression of other surface markers on CD3^+^ T cells **(A)** or LAG-3^+^ T cells **(B)** in FL. **(C)** SPADE analysis showing LAG-3 expression on different T cell populations (box) from a representative FL patient (n=28) and tonsil tissue (n=6). These T cell populations were defined based on expression of linage specific markers.

Using mass cytometry (CyTOF), we further phenotyped intratumoral LAG-3-expressing T cells as CyTOF is capable of detecting more than 40 parameters per cell. Using the viSNE map, we were able to visually and globally define LAG-3^+^ T cells in FL. As shown in Figure 3A, intratumoral CD3^+^ T cells were visually separated based on CD4, CD8, PD-1, CD25 and other markers (not shown) by viSNE analysis. Following this analysis, we performed viSNE analysis on CD3^+^LAG-3^+^ T cells from biopsy specimens of FL. As shown in Figure 3B, intratumoral LAG-3^+^ T cells were phenotypically heterogeneous as LAG-3 was expressed on a variety of types of T cell subsets including regulatory T cells (CD4^+^CD25^+^), T_H_1 (CXCR3^+^CCR5^+^), memory type T cells (CD45RO^+^). LAG-3 expression on CD127^+^ T cells suggested that some intratumoral LAG-3^+^ T cells had undergone terminal differentiation as CD127 expression typically represents the subpopulation of terminally differentiated cells. This supports the findings by flow cytometry showing that intratumoral LAG-3^+^ T cells were highly represented in TEMRA cells (CD45RA^+^CCR7^-^), a population of terminally differentiated T-cells.

SPADE is a visualization tool for high-dimensional data that includes data acquired by CyTOF. Using SPADE, we analyzed and compared LAG-3^+^ T cells from FL biopsy specimens and tonsil tissue. As shown in Figure 3C, we found that LAG-3 was expressed on a variety of types of intratumoral T cells, which is consistent with the finding by viSNE map analysis. While the number of CD8^+^ T cells was significantly reduced, LAG-3 expression by CD8^+^ T cells was negligible in tonsil. In contrast, while LAG-3^+^ T cells exhibited the similar phenotype in FL biopsy specimens to tonsil tissue (Figure 3C), the number of CD8^+^LAG-3^+^ cells was increased in lymphoma biopsies.

### The function of intratumoral LAG-3^+^ T cells is reduced

**Figure 4:**
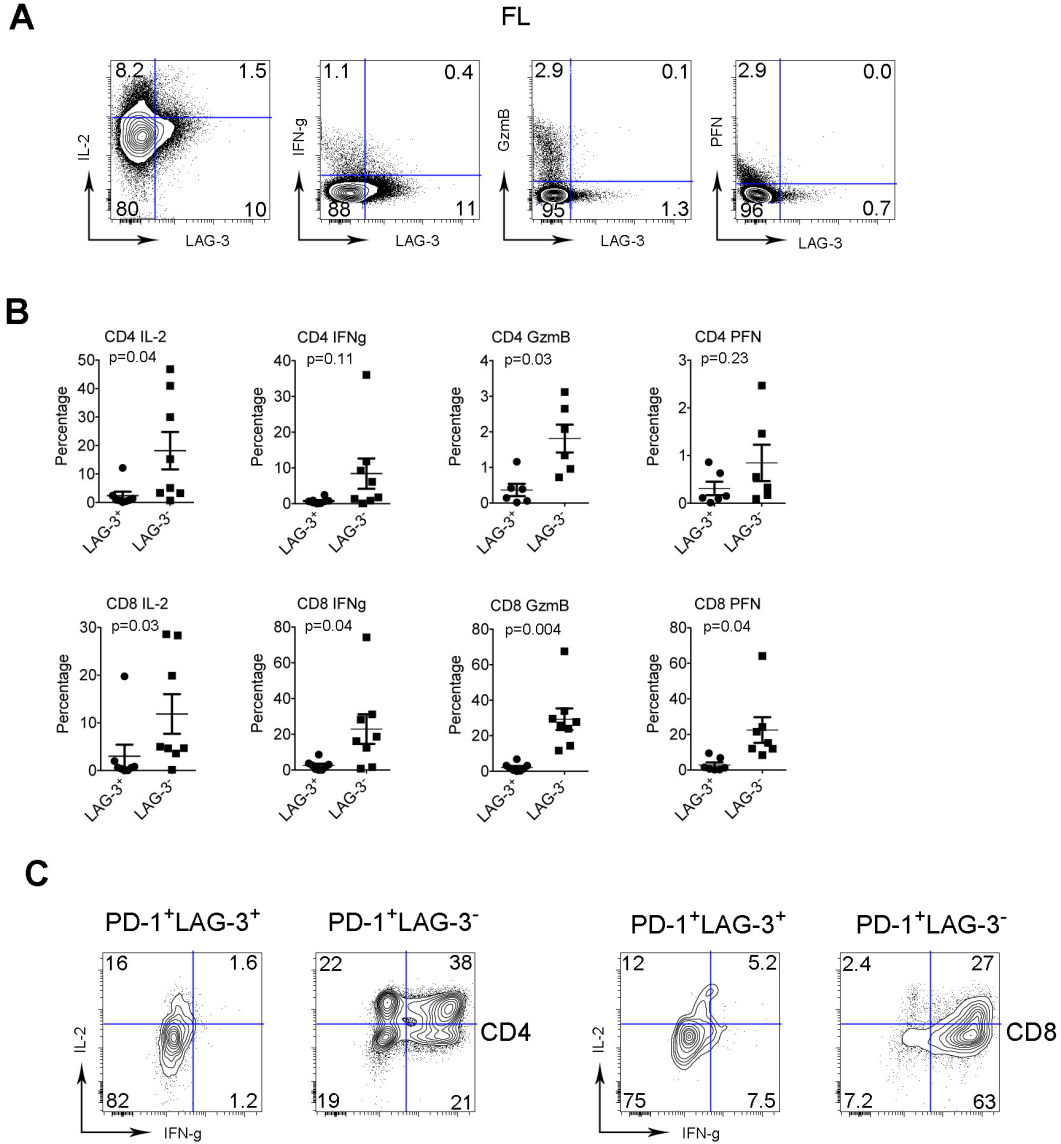
The function of intratumoral LAG-3^+^ T cells is reduced **(A)** Co-staining of IL-2, IFN-γ, GzmB and PFN with LAG-3 in intratumoral T cells from FL patients. **(B)** Graphs summarize percentages of cytokine- (IL-2 and IFN-γ) and granule- (GzmB and PFN) producing CD4^+^ or CD8^+^ T cells by LAG-3^-^ or LAG-3^+^ subset from FL patients. **(C)** Expression of IL-2 and IFN-γ by PD-1^+^LAG-3^-^ or PD-1^+^LAG-3^+^ T cells in CD4 or CD8 subset. PD-1^+^LAG-3^-^ or PD-1^+^LAG-3^+^ T cells were defined by gating co-staining of PD-1 and LAG-3.

To functionally characterize LAG-3^+^ T cells, we measured the capacity of intratumoral LAG-3^+^ T cells to produce cytokines (IL-2 and IFN-γ) and granules (perforin (PFN) and granzyme B (GzmB)) in FL. To do this, we freshly isolated mononuclear cells (MNCs) from biopsy specimens of lymphoma and stimulated cells with PMA/Ion in the presence of brefeldin A for 4 hours. The cytokine production was determined using an intracellular staining assay. For granule production, MNCs were subjected to intracellular staining assay without PMA/Ion stimulation. As shown in Figure 4A, we found that there was less cytokine and granule production by LAG-3^+^ T cells than LAG-3^-^ T cells. The numbers of cytokine-producing cells were significantly higher in LAG-3^-^CD4^+^ (mean: 18.2% and 8.39% for IL-12 and IFN-γ, respectively, n=8) or CD8^+^ T cells (11.9% and 22.9% for IL-2 and IFN-γ, respectively, n=8) compared with LAG-3^+^CD4^+^ (2.35% and 0.72% for IL-2 and IFN-γ, respectively, n=8) or CD8^+^ T cells (3.03% and 2.54% for IL-2 and IFN-γ, respectively, n=8) (Figure 4B). Similarly, the numbers of granule-producing CD8^+^ T cells were significantly higher in LAG-3^-^ (22.5% and 29.3% for PFN and GzmB, respectively, n=8) than LAG-3^+^ (2.84% and 2.14% for PFN and GzmB, respectively, n=8) T cells. Although CD4^+^ T cells normally produce very few granules, the numbers of CD4^+^ T cells producing residual granules were higher in LAG-3^-^ subset than LAG-3^+^ subset (Figure 4B). These results suggest that T cells constitutively expressing LAG-3 have reduced function.

Given that LAG-3 is primarily expressed on PD-1^+^ T cells, we wondered whether LAG-3 expression was able to differentiate PD-1^+^ subsets with differing cytokine production. To do this, we determined the expression of IL-2 and IFN-γ by subsets of PD-1^+^LAG-3^-^ or PD-1^+^LAG-3^+^ T cells. As shown in Figure 4C, while LAG-3^+^ T cells were able to produce only a modest amount of cytokines, LAG-3^-^ T cells were mainly responsible for cytokine production by PD-1^+^ subset. The numbers of cytokine-producing PD-1^+^ T cells were markedly higher in the LAG-3^-^ subset when compared to the LAG-3^+^ subset.

### LAG-3 expression is upregulated and maintained by IL-12

**Figure 5:**
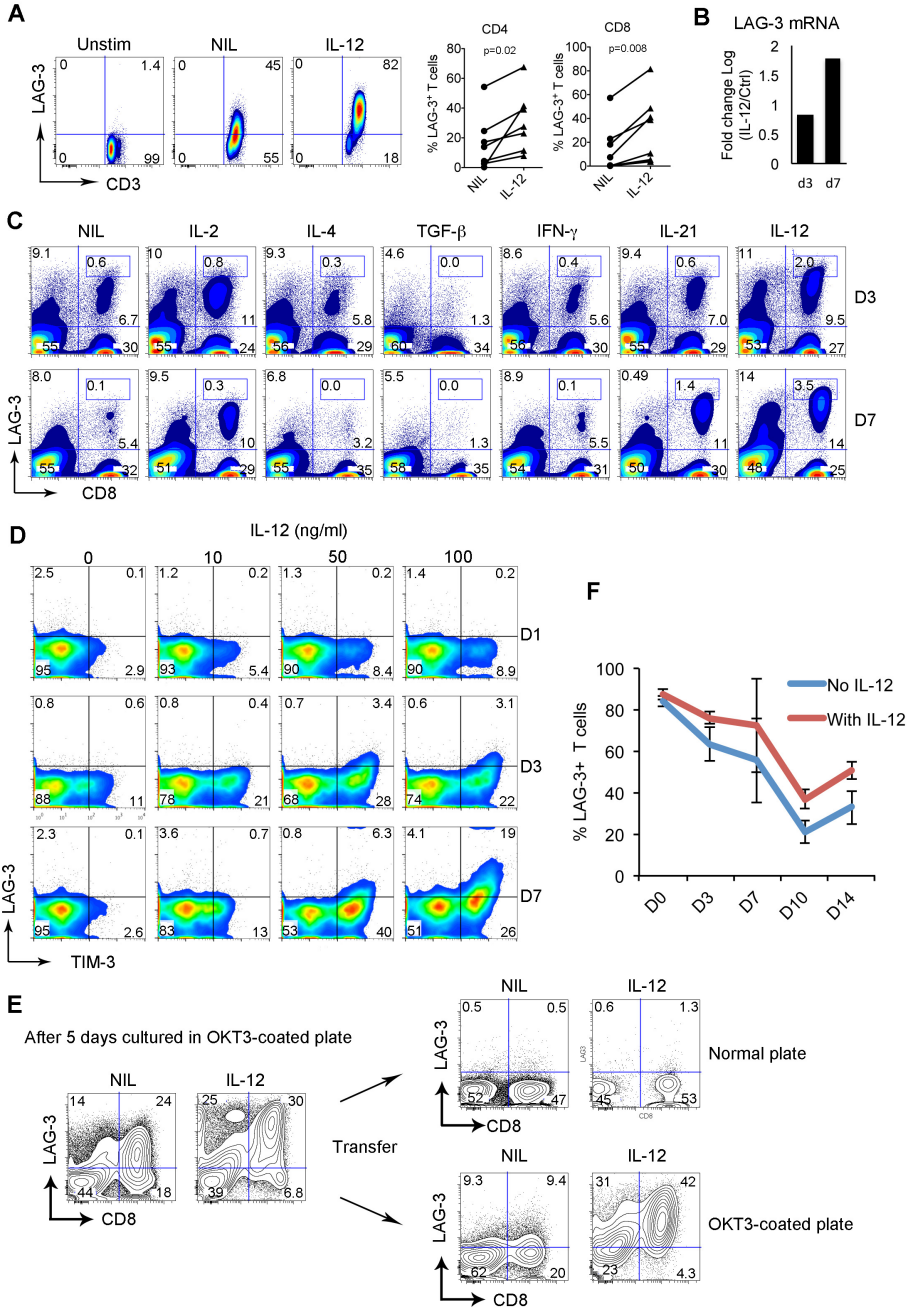
LAG-3 expression is upregulated and maintained by IL-12 **(A)** LAG-3 expression on T cells cultured in anti-CD3 Ab-coated plate with anti-CD28 Ab in the presence or absence of IL-12 (50 ng/ml) for 3 days. Cells cultured in normal plate were used as a control. The right graphs summarize percentages of LAG-3^+^CD4^+^ or CD8^+^ T cells treated with or without IL-12 measured by flow cytometry. **(B)** LAG-3 mRNA expression in CD8^+^ T cells treated with or without IL-12 for 3 or 7 days. The results were expressed as log fold change of IL-12 treated vs untreated. **(C)** LAG-3 expression on T cells cultured in anti-CD3 Ab-coated plate with anti-CD28 Ab in the presence or absence of indicated cytokines for 3 and 7 days. **(D)** Co-expression of LAG-3 and TIM-3 on CD8^+^ T cells cultured in anti-CD3 Ab-coated plate with anti-CD28 Ab in the presence of escalated doses of IL-12 for indicated days. **(E)** CD8^+^ T cells were cultured in anti-CD3 Ab-coated plate with anti-CD28 Ab in the presence or absence of IL-12 for 5 days. A portion of cells was transferred into a normal plate for 7 days. Another portion of cells remained in the anti-CD3 Ab-coated plate for the same period. LAG-3 expression was measured on day 7. **(F)** Time-course measurement of LAG-3 expression on CD8^+^ T cells pre-treated with or without IL-12 after activation was withdrawn by transferring CD8^+^ T cells into normal plate. LAG-3 expression was measured on indicated days, n=3.

Serum levels of IL-12 are elevated in lymphoma patients and are associated with T-cell exhaustion [3] and we wondered whether IL-12 played a role in the regulation of LAG-3 expression on T cells. Freshly isolated T cells from peripheral blood of healthy individuals were cultured in an anti-CD3 Ab-coated plate in the presence or absence of IL-12 (50 ng/ml) for 3 days, and LAG-3 expression was detected by flow cytometry. As shown in Figure 5A, LAG-3 expression was absent in resting T cells and activation upregulated LAG-3 expression. When treated with IL-12, LAG-3 expression was further upregulated on T cells. Supporting this finding, the mRNA level of LAG-3 was also increased on T cells treated with IL-12 (Figure 5B).

We next tested whether other cytokines could be involved in upregulating LAG-3 expression on T cells. To do this, we cultured CD8^+^ T cells in an anti-CD3 Ab-coated plate with anti-CD28 Ab in the presence of a number of cytokines for 3 and 7 days. As shown in Figure 5C, among cytokines tested, IL-4, TGF-β and IFN-γ did not increase LAG-3 expression, and TGF-β downregulated LAG-3. Treatment with IL-2 and IL-21 resulted in an increase in the numbers but not in the expression level of LAG-3. IL-12 was the only cytokine that not only strongly induced the expression level of LAG-3, but also increased the number of CD8^+^ T cells expressing LAG-3. This was particularly true when CD8^+^ T cells were treated with IL-12 for a long time period.

Given that LAG-3 was coexpressed with TIM-3 on intratumoral T cells from FL specimens, we wondered whether LAG-3 was co-upregulated with TIM-3 by IL-12 and whether the expression occurred at the same time. To do this, we cultured CD8^+^ T cells in suboptimal dose (1μg/ml) of anti-CD3 Ab-coated plate with anti-CD28 Ab in the presence or absence of IL-12 and measured expression of LAG-3 and TIM-3 on different days. As shown in Figure 5D, while TIM-3 expression was induced by IL-12 at an early time point starting on day 1, LAG-3 induction occurred at a relatively late time point starting on day 3. Interestingly, we observed that IL-12-mediated LAG-3 induction was almost exclusively on TIM-3-expressing T cells while TIM-3 induction appeared independent of LAG-3 as the majority of TIM-3^+^ T cells did not have LAG-3 expression. These results suggest that LAG-3 expression is co-upregulated with TIM-3 by IL-12 but that the expression of these two receptors is somewhat independent of each other.

Given elevated serum levels of IL-12 and the existence of T cells constitutively expressing LAG-3 in FL, we wanted to test whether IL-12 played a role in maintaining LAG-3 expression on T cells. We cultured CD8^+^ T cells in an anti-CD3 Ab-coated plate with anti-CD28 Ab in the presence or absence of IL-12 for 5 days and transferred half of the cells into a normal plate for 7 days. The other half of the cells remained in the anti-CD3 Ab-coated plate for the same period. LAG-3 expression was measured on day 7. As shown in Figure 5E, initial culture with IL-12 up-regulated LAG-3 expression on CD8^+^ T cells. After withdrawing activation by transferring cells to a normal plate, LAG-3 expression was not maintained regardless of the presence of IL-12. However, the cells remaining in the anti-CD3 Ab-coated plate maintained LAG-3 expression in the presence of IL-12 with a reduction of LAG-3 expression on CD8^+^ T cells in the absence of IL-12. We performed a time-course measurement of LAG-3 expression on T cells treated with or without IL-12 and observed that LAG-3 expression was higher on IL-12-treated T cells than IL-12-untreated T cells after activation was withdrawn (Figure 5F). These results suggest that both TCR activation and IL-12 contribute to the maintenance of LAG-3 expression on T cells.

### LAG-3 and PD-1 blockade reverses the function of T cells

**Figure 6:**
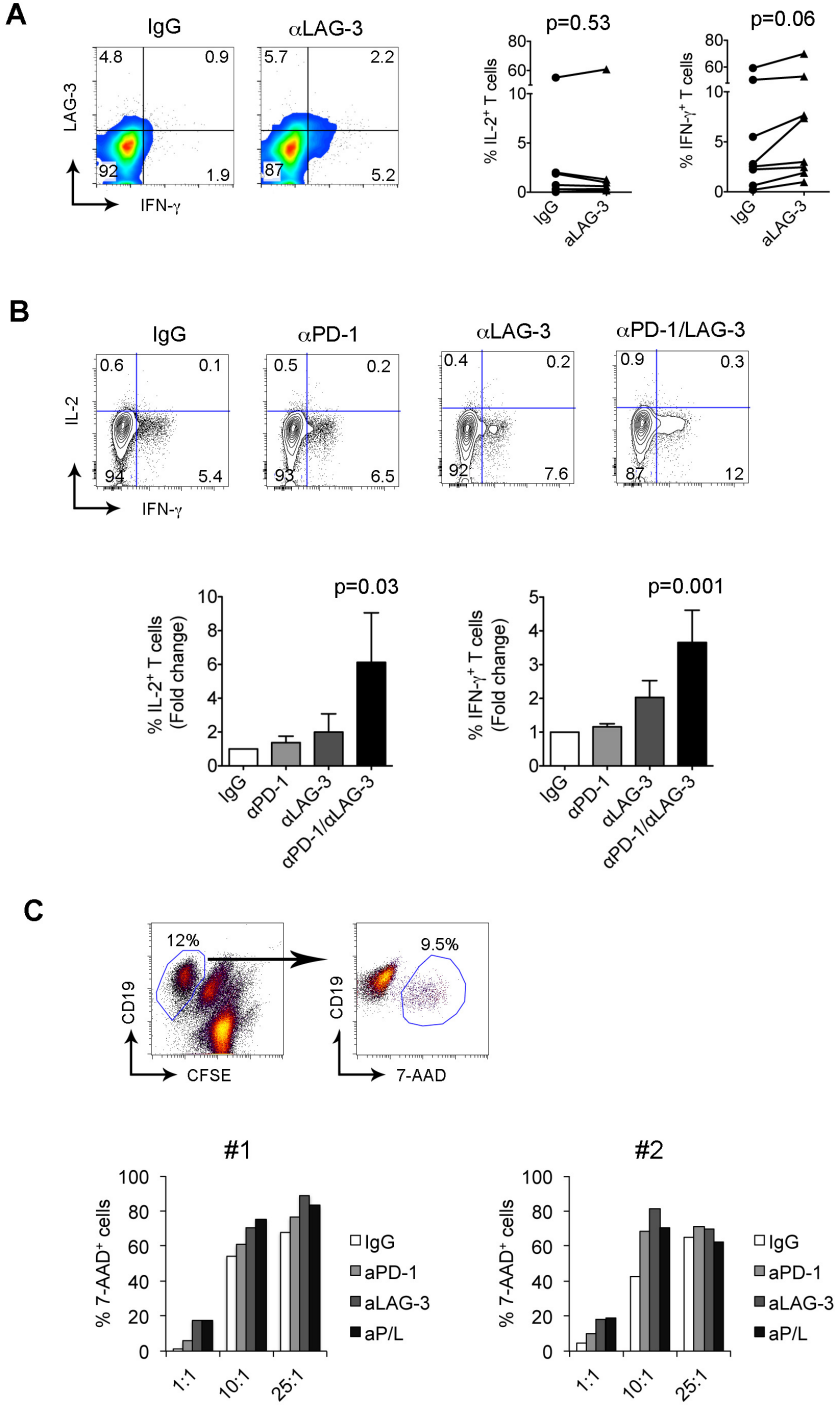
LAG-3 and PD-1 blockade reverses the dysfunction of T cells **(A)** IL-2 and IFN-γ production by CD8^+^ T cells treated with IgG or anti-LAG-3 Ab. The right graphs summarize the percentages of IL-2- or IFN-γ-producing CD8^+^ T cells treated with IgG or anti-LAG-3 Ab. **(B)** IL-2 and IFN-γ production by CD8^+^ T cells treated with IgG, anti-PD-1 and anti-LAG-3 Ab alone or in combination. The graphs below summarize the percentages of IL-2- or IFN-γ-producing CD8^+^ T cells treated with IgG, anti-PD-1 and anti-LAG-3 Ab alone or in combination. The results were expressed as fold change over IgG-treated group, n=8. **(C)** Cytotoxic activity, measured by percentage of CD19^+^7-AAD^+^ cells, of CD8^+^ T cells pre-treated with IgG, anti-PD-1 and anti-LAG-3 Ab alone or in combination toward DoHH2 cells at the indicated E/T ratios. Upper panel: 7-AAD^+^ cells were identified from target cells of CD19^+^CFSE^-^ cells. Lower panel: The percentage of 7-AAD^+^ cells was measured by flow cytometry in two samples with a total of four samples tested.

We have shown that intratumoral LAG-3^+^ T cells in FL display reduced cytokine and granule production. We next wanted to determine whether blockade of LAG-3 signaling restored T cell dysfunction. To do this, we measured the cytokine production by CD8^+^ T cells treated with an anti-LAG-3 Ab at day 7. As shown in Figure 6A, we saw an increase in cytokine production (IL-2 and IFN-γ) in CD8^+^ T cells in this particular sample. When multiple samples were tested, we observed a modest effect of LAG-3 Ab treatment on cytokine production by CD8^+^ T cells (Figure 6A). Given co-expression of LAG-3 and PD-1 on T cells, we then assessed whether dual blockade of LAG-3 and PD-1 enhanced cytokine production by CD8^+^ T cells. As shown in Figure 6B, we saw an increased production of cytokines, especially IFN-γ, in T cells treated with combination of anti-LAG-3 and PD-1 Abs at day 7. Treatment with anti-PD-1 or anti-LAG-3 Ab alone produced a negligible effect on cytokine production by CD8^+^ T cells. Interestingly, we observed that this effect was significant and highly consistent when cells were treated with LAG-3 and PD-1 blocking Ab in combination. These results indicated that dual blockade of LAG-3 and PD-1 signaling pathway restores T cell function more efficiently than either one alone.

Because Ab blockade enhances the cytokine production of CD8^+^ T cells, we next wanted to determine the effect of Ab blockade on cytotoxicity of CD8^+^ T cells when exposed to lymphoma B cells. We pre-cultured CD8^+^ T cells in anti-CD3 Ab-coated plates with anti-CD28 Ab in the presence of anti-PD-1 and anti-LAG-3 Ab alone or in combination for 7 days. Cells treated with IgG were used as a control. Pre-treated CD8^+^ T cells were co-cultured with target cells DoHH2 at E/T ratios of 1:1, 10:1 and 25:1. The cytotoxicity was determined after 3-5 days of coculture using a flow cytometry-based method. As shown in the upper panel of Figure 6C, 7-AAD^+^ cells were identified and separated from the remaining CD19^+^ DoHH2 cells when cocultured with activated CD8^+^ T cells. Using this assay, we observed dose-dependent (increased effector to target cell ratio) cytotoxicity of DoHH2 cells by IgG-treated CD8^+^ T cells (Figure 6C, lower panel). CD8^+^ T cells treated with anti-PD-1 or anti-LAG-3 increased the cytotoxic activity against DoHH2 cells when compared to IgG-treated CD8^+^ T cells. A further enhancement of cytotoxicity by CD8^+^ T cells treated with both anti-PD-1 and anti-LAG-3 antibodies together however was not consistently seen as the majority of target cells were 7-AAD positive and a further increase was difficult to demonstrate. We measured expression of HLA class I on DoHH2 cells and found that DoHH2 cells have no expression of HLA-class I, suggesting that the cytotoxic effect is independent on HLA-class I.

### Increased numbers of LAG-3^+^ T cells correlate with poor outcome in FL

**Figure 7:**
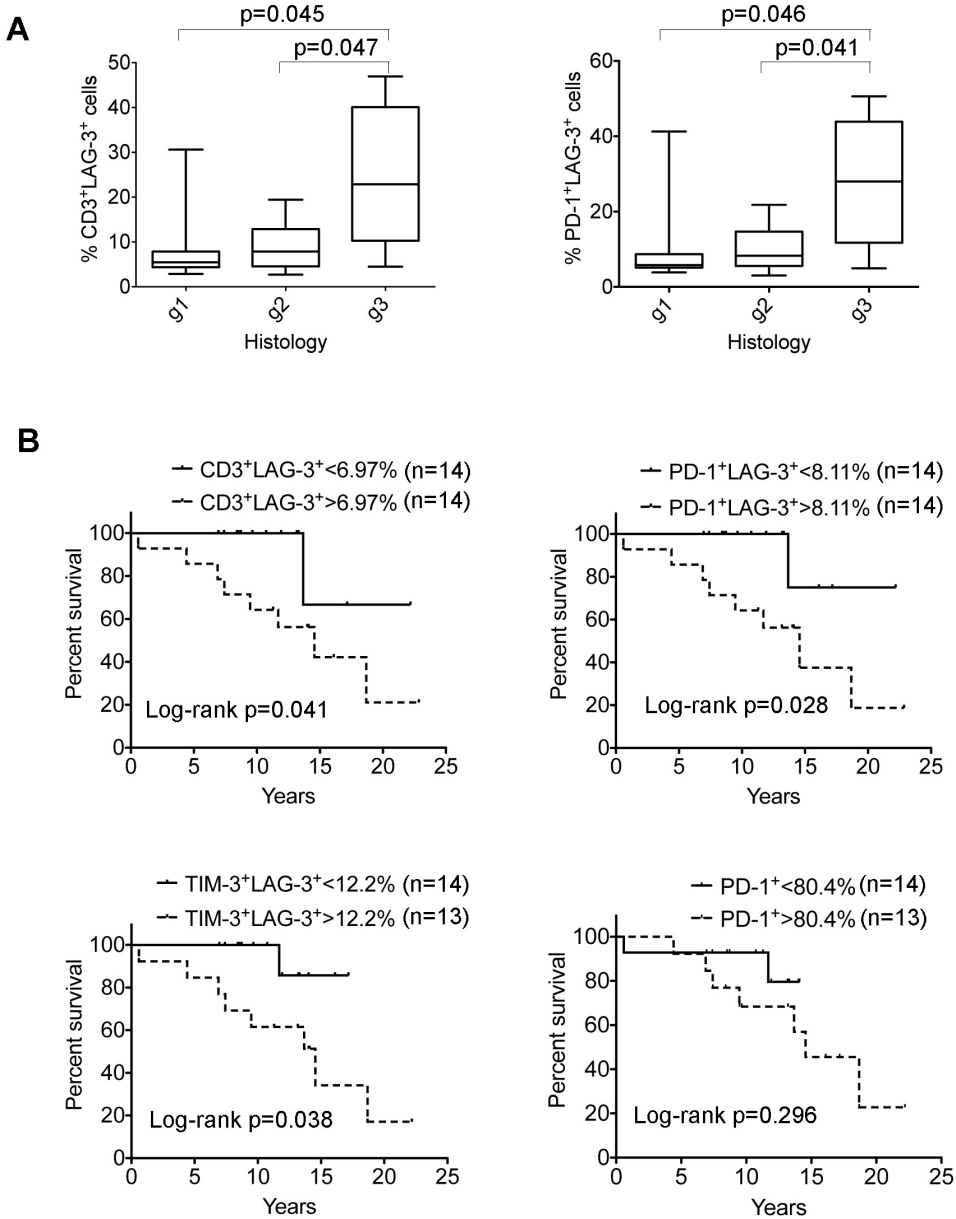
Correlation of LAG-3–expressing cells with clinical survival in FL patients **(A)** Correlation of the numbers of CD3^+^LAG-3^+^ cells (left) or PD-1^+^LAG-3^+^ (right) T cells with histological grade (g1-3) in FL patients (n = 28). **(B)** Kaplan-Meier curves for overall survival of FL patients (n = 28) by the number of CD3^+^LAG-3^+^ cells (upper left), PD-1^+^LAG-3^+^ T cells (upper right), TIM-3^+^LAG-3^+^ T cells (lower left) or PD-1^+^ T cells (lower right) with a cutoff of 6.97%, 8.11%, 12.2% or 80.4%, respectively.

Given that lymphoma-associated LAG-3^+^ T cells were functionally exhausted, we predicted that intratumoral LAG-3^+^ T cells would adversely affect patient outcomes in FL. Using tumor samples from 28 FL patients, we found that LAG-3^+^ T cells accounted for a median of 6.97% (range: 2.7%- 46.9%) of intratumoral CD3^+^ T cells and 8.11% (range: 3.02%-50.6%) of intratumoral PD-1^+^ T cells. We observed that increased numbers of CD3^+^LAG-3^+^ cells or PD-1^+^LAG-3^+^ T cells were associated with a higher histological grade (Figure 7A) in FL patients. Furthermore, the frequency of CD3^+^LAG-3^+^ cells as well as TIM-3^+^LAG-3^+^ T cells significantly correlated with poorer survival in FL patients (Figure 7B). While total PD-1^+^ T cells had no impact on patient outcome, the numbers of LAG-3-expressing PD-1^+^ (PD-1^+^LAG-3^+^) T cells were associated with poor survival in FL patients (Figure 7B), suggesting that LAG-3 expression plays an important role in defining exhausted PD-1^+^ T cells in FL.

## DISCUSSION

The significant findings in the present study are: first, PD-1^+^, and not PD-1^-^ T cells are mainly responsible for the production of cytokines and granules in FL suggesting that PD-1 expression is associated with both activation and exhaustion; second, LAG-3 is primarily expressed on PD-1^+^ and TIM-3^+^ T cells, forming a subset of PD-1^+^TIM-3^+^LAG-3^+^ CD4^+^ or CD8^+^ T cells that are associated with a poor prognosis; third, the function of LAG-3^+^ T cells is significantly reduced and constitutive expression of LAG-3 functionally differentiates PD-1^+^ T cells into those with retained function and those that are exhausted, and fourth, dual blockade of PD-1 and LAG-3 in combination is able to reverse T cell function more efficiently than blocking either signaling pathway alone.

PD-1 expression is typically used to identify exhausted T-cells in patients with chronic viral infection. However, we have previously shown that, in FL, not all PD-1^+^ cells are exhausted and that PD-1 is differentially expressed on two distinct T-cell subpopulations, with high expression on T follicular helper cells and dim expression on exhausted T cells. In this study, we have shown that PD-1^low^ T cells, a T-cell population that is expected to be exhausted, are mainly responsible for the production of cytokines and granules in FL. This finding appears to be contrary to the findings in chronic viral infections where PD-1^+^ T cells have reduced function. Our results suggest that these PD-1^+^ T cells are functionally heterogeneous and other markers are needed to define the function of PD-1^+^ T cells.

This surprising finding prompted us to explore the underlying mechanisms by which PD-1^+^ T cells mediate T-cell exhaustion. LAG-3, a member of the immunoglobulin superfamily, has been shown to play a negative regulatory role in T cell-mediated immune responses. Previous studies have found that LAG-3 is coexpressed with PD-1 on T cells and contributes to the development of exhaustion in chronic viral infections [17, 18] and tumors [19, 20]. In this study, we specifically find that LAG-3 is coexpressed on PD-1^+^ T cells, thereby defining a population of cells that phenotypically appear exhausted [5].

We found that LAG-3 expression on T cells is upregulated by IL-12, which is in keeping with previous reports [21, 22]. Although other cytokines such as IL-2 and IL-21 are also involved in the upregulation of LAG-3 expression, IL-12 remains to be the only cytokine tested that both upregulates the expression of LAG-3 and increases the number of T cells expressing LAG-3. After upregulation, the maintenance of LAG-3 expression requires activation as well as IL-12 stimulation. Given elevated serum levels of IL-12 [3], this result may provide an explanation for the increased numbers of LAG-3^+^ T cells in FL patients. The finding that LAG-3 and TIM-3 were co-upregulated by IL-12 may explain the existence of the TIM-3^+^LAG-3^+^ T cell population in FL.

We find that the function of LAG-3-expressing T cells is reduced and the numbers of T cells producing cytokines and granules are significantly lower in the LAG-3^+^ subset compared to LAG-3^-^ T cells. We show that expression of LAG-3 differentiates functionally different populations of PD-1^low^ T cells. We find that LAG-3^-^, but not LAG-3^+^, T cells are predominantly responsible for cytokine and granule production within the PD-1^low^ T cell population. Although other studies have found co-expression of PD-1 and LAG-3, our data clearly shows a key role for LAG-3 in PD-1-mediated T cell exhaustion. Another finding to support the important role of LAG-3 in PD-1-mediated exhaustion of T cells is that functional reversal of T cell exhaustion required dual blockade of LAG-3 and PD-1. Blockade with anti-PD-1 or anti-LAG-3 Ab individually produces marginal effects. Dual blockade with the combination of anti-PD-1 and LAG-3 Abs results in a significant and consistent effect in terms of increased cytokine production by intratumoral T cells in FL, which is consistent with previous reports [19, 23, 24]. While PD-1 ligands are broadly expressed on monocytes/macrophages, B cells, endothelial cells and tumor cells, PD-1 ligands are also expressed on activated T cells [25] and intratumoral T cells [26]. Therefore, blockade of PD-1 and LAG-3 ligands expressed on activated T cells in these co-culture systems upregulated cytotoxic T-cell function.

In addition to its biologic role in T-cell exhaustion, LAG-3 is clinically relevant in impacting patient outcome in FL. We observed that LAG-3 expression on intratumoral T cells correlated with a poor outcome in FL patients. Consistent with our findings, previous studies showed that patients with positive or high density of LAG-3 had a poor survival in renal cell cancer and non-small cell lung cancer [27, 28]. Notably, we found that LAG-3 expression identified a subpopulation of PD-1^+^ T cells that impact patient outcome. We found that increased PD-1^+^LAG-3-producing T cells were associated with an inferior patient survival, while the total population of PD-1^+^ T cells did not predict patient outcome in FL. This result suggests that LAG-3 expression defines the non-functional, and therefore poor prognostic, PD-1^+^ T cell population in FL.

In summary, we present data in the present study showing that LAG-3 expression in lymphoma tissue specifically identifies the cells within the PD-1^+^ T cell population that are functionally exhausted. In contrast, intratumoral PD-1^+^LAG-3^-^ T cells are in fact activated cells that retain their ability to produce cytokines when stimulated. Furthermore, we find that dual Ab blockade of the PD-1 and LAG-3 pathways restores T cell function more efficiently than either one alone. Clinically, LAG-3^+^ T cells, particularly PD-1^+^LAG-3^+^ T cells, predict a poor outcome in patients with FL. These results may be very important as we determine which population of cells to target with immune checkpoint blockade. Simply blocking PD-1 alone may well reinvigorate exhausted T-cells, but may also affect activated cells. Rather, dual blockade of both PD-1 and LAG-3 may specifically target the exhausted T-cell population and may therefore be a promising future treatment approach in lymphoma patients.

## MATERIALS AND METHODS

### Patient samples

Patients providing written informed consent were eligible for this study if they had a tissue biopsy that on pathologic review showed FL and adequate tissue or peripheral blood to perform the experiments. Peripheral blood mononuclear cells from healthy donors and lymph nodes from patients with hyperplasia were used as controls. The use of human tissue samples for this study was approved by the Institutional Review Board of the Mayo Clinic/Mayo Foundation.

### Cell isolation and purification

Fresh tumor biopsy specimens from patients with FL and normal lymph nodes were gently minced over a wire mesh screen to obtain a cell suspension. The cell suspension or peripheral blood from patients or healthy donors was centrifuged over Ficoll Hypaque at 500 x g for 15 minutes to isolate mononuclear cells. CD4, CD8^+^ T cells, or CD19^+^ B cells were isolated using positive selection with CD4, CD8 or CD19 microbeads (StemCell Technologies, Vancouver, Canada).

### Intracellular staining and flow cytometry

For profiling of cytokine production by PD-1^+^ and LAG-3^+^ CD4^+^ or CD8^+^ T cells, fresh-isolated mononuclear cells from lymphoma biopsy specimens were stimulated with phorbol myristate acetate (PMA) and ionomycin (Ion) in the presence of protein transport inhibitor Brefeldin A for 4 hours. For detection of granule (perforin and ganzyme B) production assay, stimulation with PMA/Ion was not needed. Cells were first stained with surface marker antibodies for CD4, CD8, PD-1 and LAG-3. After washing, cells were fixed, permeabilized and stained with fluorochrome-conjugated antibodies for IL-2 and IFN-γ as well as perforin and granzyme B. Cells were analyzed on an 8-color Canto flow cytometer.

### Mass cytometry (CyTOF)

CyTOF assay was performed according to the manufacturer’s instruction. Briefly, 3×10^6^ cells were stained with 5μM Cell-ID™ Cisplatin (Fluidigm, San Francisco, CA) for 5min and quenched with MaxPal Cell Staining Buffer (Fluidigm) using 5× the volume of the cell suspension. After centrifugation, cell suspensions (50μl) were incubated with 5μl of human Fc-receptor Blocking solution (Biolegend, San Diego, CA) for 10min and 50μl of pre-mixed antibody cocktail for 30 min. After washing, cells were incubated with 1ml of cell intercalation solution (125 nM MaxPal Intercalator-Ir into 1ml MaxPal Fix and Pem Buffer, Fluidigm) overnight at 4°C. Cells were centrifuged with MaxPal Water and pelleted. The pelleted cells were suspended with EQ Calibration Beads (Fluidigm) and cell events were acquired by a CyTOF II instrument (Fluidigm). The data were analyzed using online software Cytobank, viSNE map and SPADE.

### Cytotoxicity assay

A flow-based cytotoxicity assay was used to measure *in vitro* cellular cytotoxicity of CD8^+^ T cells against lymphoma B cells as previously described [29, 30]. CD8^+^ T cells were cultured in anti-CD3 Ab-coated plates with anti-CD28 Ab in the presence of anti-PD-1 and anti-LAG-3 Ab alone or in combination for 3-5 days. Cells treated with IgG were used as controls. Pretreated T cells (effector cells, E) were labeled with 250 nmol/L of CFSE and cocultured with the lymphoma B-cell line DoHH2 (target cells, T) at different E:T ratios. In parallel, target cells were incubated alone to measure basal apoptosis. Immediately before analysis, 1 μg/mL (final concentration) of 7-amino-actinomycin D (7-AAD; Calbiochem, La Jolla, CA) was added to each sample and incubated for 20 minutes at 4°C in the dark. Cells were then harvested and analyzed by flow cytometry to measure the percentage of CD19^+^7-AAD^+^ cells.

### Statistical analysis

Statistical analysis was performed using Student's t test. Significance was determined at p< 0.05. Overall survival was measured from the date of diagnosis until death from any cause. Patients alive and still at risk of death at last follow-up evaluation were censored for the analysis of overall survival. Survival of all patients was estimated using the Kaplan-Meier method. The univariate association between LAG-3 expression and survival was determined with the log-rank test.
